# Review of Head and Neck Masses in the Indian Population Based on Prevalence and Etiology With an Emphasis on Primary Diagnostic Modalities

**DOI:** 10.7759/cureus.16249

**Published:** 2021-07-07

**Authors:** Nilam Bhasker

**Affiliations:** 1 Pathology, Employees' State Insurance Corporation Hospital, Lucknow, IND

**Keywords:** head and neck swellings, fnac, usg, ct, mri

## Abstract

Head and neck masses are classified as sebaceous cysts (epidermoid cysts), cervical lymphadenopathy, benign lipomas, lymph nodes, thyroid swellings, or tuberculosis lymphadenitis that may be painful or painless, adherent or fluctuant. In spite of this, they have distinct prognoses and pathological features. The anatomical location of the swelling and other demographic manifestations of the patient provide valuable information about the cause and type of swelling. Computed tomography (CT), magnetic resonance imaging, positron emission tomography-CT, and ultrasonography are the gold standard imaging methods for the head and neck examination. These methods are used according to the region considered for the study. Fine-needle aspiration cytology of lymph nodes is known to be effective, simple, and sometimes the only tool for the diagnosis of lymph node malignancies. This review highlights the epidemiological aspect of head and neck masses in the Indian population.

## Introduction and background

The presence of external superficial mass (lump or nodule) in the head region is a frequent reason for patients to seek an ear-nose-and-throat (ENT) consultation. Some of the common causes for a bump on the scalp are acne, eczema, psoriasis, pilar cysts, hives, or ringworms. Other possible etiologies may be infections, congenital lesions, benign growths, or malignant tumors. The common causative factors can be addressed at home, and in the case of inadequacy, the patient can contact a healthcare professional. Additionally, if the bump is caused by skin cancer, the patient requires urgent medical attention.

Similarly, neck masses are commonly encountered by a medical practitioner in clinical practice. A mass in this region has important implications owing to the complex anatomy of the head and neck. The presence of significant underlying structures has great physiological importance too. As a result, the causes of neck swellings may have multiple reasons, and each having a distinct pathology and prognosis while infectious swellings, lymphadenopathies, and benign and malignant neoplasms are the most common causes. Factors like the anatomic location of the swelling and the age of patients offer valuable information about the etiology and type of swelling. For instance, a swelling appearing after the age of 40 is more likely to be malignant. Furthermore, rigorous clinical evaluation along with imaging or pathological investigationslike fine-needle aspiration cytology (FNAC)/biopsy is required to make a diagnosis, to depict the form and origin of the swelling [1].

## Review

Surgeons often encounter head and neck masses in their clinical practice. Swellings in this area have serious implications due to the complex anatomy, as well as the physiological importance of the underlying structures. Furthermore, the presence of multiple structures in the neck results in a difference in the origin of the swelling, and each variety has a distinct pathology and prognosis. The most frequently occurring forms are infectious swellings, lymphadenopathies, and benign and malignant neoplasia. The anatomic location of the swelling offers valuable information about the etiology and type of swelling, as the patient ages. For example, a swelling appearing after the age of 40 is more likely to be malignant. For diagnosing a neck swelling, a rigorous clinical evaluation is required that may be accompanied by sophisticated diagnostic technologies to achieve firm evidence and certainty about the type and origin of the swelling, such as the use of imaging or pathological investigations like FNAC/biopsy to confirm a case of recurrent neck mass [1].

Imaging methods are an important part of the diagnostic process to evaluate a neck mass. Ultrasonography is the most relevant screening tool in contrast to other imaging modality and it is recommended for the initial workup due to its non-invasive nature and the absence of ionizing radiations. Subsequently, if the physical examination fails to diagnose the neck mass, then FNAC is recommended, which is preferred as the first line of inquiry in the diagnosis of head and neck swelling [1].

Sebaceous cyst

Giant sebaceous cysts are uncommon in clinical practice while they are the most common presentation in young adult males [2]. These cysts can occur at any age but do not occur before puberty. They frequently occur on the face, trunk, throat, scalp, scrotum, ear lobe, buttock, and breast, but a giant sebaceous cyst at an uncommon site raises concern [3]. These also occur in females, typically on the forehead, who work in outdoor environments with an exposure to sunlight and unhygienic conditions. The size of a usual sebaceous cyst ranges from a few millimeters to centimeters (1-4 cm in diameter) whereas if the cysts grows up to more than 5 cm, it is called a giant sebaceous cyst [4]. Small cysts normally grow at a rate of <0.5 cm/year; therefore, they take a year to grow into giant cysts eventually. Also, the rate of expansion is faster in the early years than after becoming a giant cyst. Cysts are situated intradermally and formed by rupturing of the pilosebaceous follicles. In spite of this, they are adherent to the epidermis and have an easily recognizable central punctum [2,5]. These dome-shaped lesions are usually asymptomatic, painless, and have smooth skin overlying thick sebum. Giant sebaceous cysts have a high risk of being cancerous [5]. Gardener's syndrome with associated pre-malignant colonic polyps, pachyonychia congenital, lipomas or fibromas of the skin, and osteomas should be treated as a part of Gardener's syndrome [6].

Lipoma

Lipomas are the most common benign mesenchymal neoplasms that can appear anywhere in the body where fat is present. They are advanced adipose cell tumors that mostly affect the upper back, leg, and abdomen. In the case of the head and neck, the posterior neck region is the commonest site of involvement (about 13% of lipomas are found here) [7]. The clinical features of a lipoma depend upon the size, location, and growth rate of the lesion besides the usual cosmetic considerations. A lipoma is manifested by a well-circumscribed mass that grows in size over time. The exact cause of lipoma is unknown while some potential causes are heredity, obesity, diabetes mellitus, trauma, radiation, endocrine disorders, insulin injection, and corticosteroid therapy [8]. Furthermore, lipomas are linked to inherit multiple lipomatosis, adiposis dolorosa, Gardner's syndrome, and rarely Madelung's disease [9].

Most of the lipomas can be managed without surgical treatment while surgery is required when there is diagnostic uncertainty, lack of homogeneity on palpation, a broad neck mass (>10 cm), rapid progression in size, associated discomfort or cosmetic concerns, or a deep-seated lipoma (intramuscular or intermuscular) [10]. Complete surgical excision is the most effective treatment; however, liposuction may be helpful in areas such as the face. Also, liposuction is often preferred to excision because of the minimal scarring; however, there is a higher risk of recurrence with the former [10].

Lymph node swelling

The term lymphadenopathy refers to the swelling of lymph nodes as a clinical indication of neck inflammation [11]. Cervical lymphadenopathy is a common complication of bacterial, viral, and dental infections, as well as surgical procedures of the head and neck. It can be easily diagnosed when patients have accompanying symptoms of a systemic infection, oral trauma, or dental infections. Notably, lymphadenopathy caused by an inflammatory disease normally clears up by four to six weeks; consequently, any swollen node that lasts longer than two weeks needs further investigation. On palpation, lymph nodes that are greater than 1.5 cm in diameter are solid, rubbery, or matted, and nodes that are fixed or have reduced mobility. The submandibular and submental nodes are included in the first level, while levels II, III, and IV cover lymph nodes of the upper, middle, and lower thirds of the body, respectively, and are located along the internal jugular vein deep to the sternocleidomastoid muscle. The nodes of the posterior triangle are assigned in level V.

The number of lymph nodes involved in a person's body may indicate the origin of the pathology. Some levels for lymph nodes in the head and neck and their corresponding areas of pathology are mentioned next* *[11].

Level IA: Submandibular Region

Cancers of the floor of the mouth, anterior tongue, anterior mandibular alveolar ridge, and lower lip have the highest risk of metastasis affecting the level IA nodes, while cancers of the oral cavity, anterior nasal cavity, soft tissue structures of the midface, and submandibular gland are prone to metastasize to the level IB nodes.

Level IIA and IIB: Upper Jugular Group

Cancers of the oral cavity, nasal cavity, nasopharynx, oropharynx, hypopharynx, larynx, and parotid gland are highly susceptible to metastasizing to the level II nodes.

Level III: Middle Jugular Group

Cancers of the oral cavity, nasopharynx, oropharynx, hypopharynx, and larynx are the most common sources of metastasizing to the level III nodes.

Level IV: Lower Jugular Group

These metastasize from cancers of the larynx, hypopharynx, thyroid, and cervical esophagus.

Levels VA and VB: Posterior Triangle Group

Here, there are high chances of metastasis from the cancers of the nasopharynx, oropharynx, and skin of the posterior scalp and neck.

Level VI: Central Compartment Group

These nodes are at the highest risk of harboring metastasis from cancers arising from the thyroid gland, glottis and subglottic larynx, apex of the pyriform sinus, and cervical esophagus [11].

Thyroid nodule

A thyroid nodule is described as a single lesion within the thyroid gland that is palpable and/or ultrasonographically distinct from the surrounding thyroid parenchyma [12,13]. Thyroid nodules are extremely common; about 50% of adults having thyroid nodules can be confirmed on ultrasound, and 7% of the affected adults have palpable nodules [14]. Malignant thyroid nodules account for around 5% of all thyroid nodules. Nodules in the thyroid gland may occur alone, sometimes encountered as an incidental finding, or can present with associated symptoms of systemic thyrotoxicosis or hypothyroidism. They may exist as a single nodule or as multiple nodules in multinodular goiter [15]. The incidence of thyroid cancer is comparable in patients with a solitary nodule and multiple nodules; solitary nodules are at a higher risk of transforming into malignant [16]. Thyroid nodules are more common in women, the elderly, people with iodine deficiency, and those who have been exposed to radiation in the past. The majority of people with thyroid nodules do not have any noticeable signs and symptoms; however, a lack of signs does not rule out the possibility of cancer. If thyroid swelling is symptomatic, certain factors, such as the duration of symptoms, the speed at which they shift, the presence of systemic hyper- or hypothyroidism, and a family history of cancer, should be investigated [17].

The probability of the nodule to transform into malignant increases in the presence of following features: (1) extremes of age (70 years); (2) male gender; (3) size of nodule >4 cm; (4) rapid growth (maybe suggestive of anaplastic carcinoma or lymphoma); (5) family history of thyroid malignancy or genetic disorders (such as syndrome of multiple endocrine neoplasia [MEN] type 2, familial polyposis coli, or Cowden syndrome); (6) prior exposure to radiation, including radiotherapy to the head and neck (e.g., previous treatment of Hodgkin’s disease affecting the neck); (7) a painful thyroid nodule that may be a sign of subacute thyroiditis (single or multiple); most thyroid nodules are painless and the presence of pain may be indicative of the cause of the nodule. Additional indications for biopsy include an inability to obtain an adequate sample by FNAC after several attempts, or when a nodule with malignant features is seen on ultrasonography [18].

Salivary glands

Swelling in the salivary glands is an unusual occurrence in the head and neck region in terms of the frequency, variability, and peculiarity of its pathological process. Several salivary glands are present in the lips, cheeks, mouth, and throat. Tumorous growths can develop in any of these glands, but salivary gland tumors are most frequently found in the parotid glands; the majority of these parotid tumors are benign whereas some may transform into malignant. Numbness, burning, or prickling sensations in the ears, or a lack of facial expression are some common symptoms of parotid swelling while the tumors may also present as a non-painful swelling in the face or jaw. Usually, the first step of management for this tumor is surgical removal that can be prescribed as adjunct if the tumor is malignant in nature. Since management of these tumors is associated with the risk of developing a fistula and tumor implantation in the case of neoplasm, they are rarely exposed to incisional or needle biopsy techniques. Cytology studies can help to distinguish between parotid/non-parotid lesions, benign/malignant lesions, and acute/non-severe inflammation; consequently, a definitive course of action for the therapeutic management of the patient can be planned. FNAC is a useful tool for subtyping parotid gland lesions; however, its accuracy and sensitivity are variable. AlGhamdi et al. reported overall sensitivity and specificity for salivary gland masses as 90.3% and 100%, respectively. The positive predictive value, negative predictive value, and diagnostic accuracy were 100%, 57.1%, and 91.4%, respectively [19].

Nodal tuberculosis

Tuberculosis (TB) continues to be *endemic *in many parts of the world including India; therefore, meticulous surveillance, thorough clinical assessment and testing, contact tracing, and confirmation of diagnosis are essential for treatment regimens and effective eradication of the disease [20]. In the case of TB infection, females outnumber males in terms of the gender distribution of the disease [21]. Earlier, India accounted for about 26% of TB cases in the world. However, TB notifications were reduced by 25% between January 2020 and June 2020 in India compared to the same period in 2019 due to an increase in screening programs [22]. FNAC reportedly has a diagnostic accuracy of 90%-100% in detecting TB affliction of the regional lymph nodes. Culture, cytological analysis of needle aspirates with Ziehl-Neelsen (ZN) staining, chest radiography, and the Mantoux test, along with meticulous history and detailed clinical inspection, are the most effective battery of tests to confirm the diagnoses or a similar expression can be used. A battery of investigation is needed to establish a confirmed diagnosis of cervical TB, especially in developing countries [21]. A culture of the lymph node aspirate is needed to detect the organism when blood smears are reported to be bacilli-negative, suspicious of TB and repeated episodes of lymphadenopathy even after anti-tubercular treatment. The cytological presentation of tubercular lymphadenitis may be confused with reactive hyperplasia due to viral or toxoplasma infection, especially in the case of infants, where the aspirate represents a polymorphous picture with occasional epithelioid cells. An open biopsy is required for clarification when the Langhans giant cells or caseous necrosis is not found during the cytological examinations. Since, at times, tubercular lymphadenitis may be the only symptom of TB and the associated constitutional characteristics may not be noticeable, it is prudent to explore an avenue of history, clinical examination, and radiological or laboratory examinations for relevant information to confirm the TB diagnosis.

Characteristics of head and neck mass that need immediate action include the following: (1) no history of any recent infection, and if so, infection is unlikely to be the etiology for the neck mass; (2) persistence of the mass for two or more weeks or uncertain duration; (3) reduced mobility of neck mass (metastatic cancer may violate the lymph node capsule, directly invading the adjacent structures); (4) firm texture of the mass (a malignant lymph node is often firm due to the absence of tissue edema; a neck mass may be soft due to its fluid content or may be due to a benign cystic mass, fluid-filled cystic masses may also be malignant, and an infectious lymph node may be soft due to tissue edema); (5) neck mass with a size >1.5 cm (lymph node metastasis resulting in nodal enlargement); and (6) ulceration of the skin overlying the neck mass. Metastatic cancer may rupture through the capsule of the lymph node and directly invade and necrose the overlying skin. Consequently, ulceration overlying a neck mass may be indicative of a cutaneous malignancy with direct extension into the neck [23].

Epidemiology of head and neck masses

India is a vast country with a heterogeneous distribution of population. The prevalence of the above-mentioned lesions greatly varies in different parts of the country, which is associated with multiple epidemiological factors. One of the studies reported that approximately 7% of all cases of epidermoid and dermoid cysts are associated with the head and neck region [24]. A recent study demonstrated that epidermoid cysts are one of the commonest cutaneous cysts, uniquely occurring in the third and fourth decades of life and rarely occur before puberty. This study also reported that they more frequently occur in males (female vs. male ratio, 2:1) compared with females, and approximately 1% of epidermoid cysts transform into malignant squamous cell carcinoma (SCC) and basal cell carcinoma (BCC) [25].

A study led by Bajpai et al. reported the prevalence of lipoma in the Indian population; the mean age of the affected patients was 41.9 ± 3.5 years after an evaluation of 53 cases of oral lipoma (16 institutional + 37 published) [26]. This study has also shown that the lesion was more common among males (58%) and found that buccal mucosa was the most frequently affected site. Histopathologically, the most common type of lipoma was a simple lipoma, followed by fibro lipoma, angiolipoma, osteolipoma, chondrolipoma, myxolipoma, spindle cell lipoma hibernoma, intramuscular lipoma, and angiomyxolipoma. The rarest variety of lipomas in the oral cavity was found to be hibernoma. Fregnani et al. conducted a study predominantly on adults with 46 subjects and reported that buccal mucosa was the most common site for lipoma, followed by the tongue, lips, and floor of the mouth. They also reported a similar occurrence pattern of lipoma in which simple lipomas were the most common, followed by fibro lipoma, intramuscular lipomas, adenolymphoma, and spindle cell lipomas [27]. Similar results were also reported by Furlong et al. who analysed a predominantly male sample with intraoral lesions [28]. Studart-Soares et al. reported that buccal mucosa and simple lipoma more frequently occur in males with a mean age of 53.4 years [29].

Several lines of evidence have been reported on the occurrence, incidence, and pervasiveness of lymphadenopathy in the Indian population [30-32]. A study conducted by Das et al. reported that approximately 47.5% of the affected patients had infective or inflammatory lymphadenopathy, followed by tubercular lymphadenopathy (33.33%), metastatic nodes (13.33%), and lymphoma (1.67%) [30]. One of the studies showed that lymph node TB accounts for 35% of extra-pulmonary TB with two-thirds of the patients having cervical lymph nodes [31]. A study performed by Sarda et al. reported the high incidence of tuberculous lymphadenitis with 86% of the swollen nodes being tuberculous in origin [32]. Conversely, some of the studies reported a low incidence of tubercular lymphadenitis in swollen neck nodes (3.5%) that can be explained by the fact that all nodes were examined regardless of the extent of node enlargement [33-36]. A recent study demonstrated that cervical lymphadenopathy was more prevalent in young and middle-aged adults, and the prevalence of cervical lymphadenopathy was found to decrease with age [37]. Abba et al. reported that females were more frequently affected by tubercular lymphadenopathy (accounting for 68% of the cases) compared to males (32%) [38]. Several authors believed that the predilection of females for tubercular lymphadenopathy may be due to their jobs, especially in the lower socioeconomic groups, as well as poor healthcare services for women in rural areas. An epidemiological review suggested that illiteracy and a lack of understanding are closely linked to a higher prevalence of extra-pulmonary TB [39]. Maharajan et al. found that the posterior triangle group of nodes was most commonly affected in cervical lymph node involvement [39]. FNAC is considered as the most reliable, responsive, and precise technique for diagnosing lymphadenopathy. Approximately 82% of the 40 cases of tubercular lymphadenopathy had epithelioid granuloma and Langhans cells, with or without necrosis, as reported in patients who underwent FNAC. The ZN stain for acid-fast bacilli was positive in 55.5% of the cases who were also subjected to cervical lymph node ultrasonography [40]. A study led by Das et al. showed that hypoechogenicity and necrosis were present in all cases (100%) while matted lymph nodes were present in 16 (40%) patients. Another study reported calcifications in 11 patients (27.5%) and sharp margins in 27 patients [30].

Thyroid disorder is another disease that is most frequent in the Indian population. Recently, the health burden of this disease has been found to increase [41]. Studies have shown that about 12% of Indian adults have a palpable goiter [42]. Autoimmune thyroid disease is probably commoner than iodine deficiency. However, given that iodine deficiency is endemic in India, the emphasis is more on iodine deficiency in the Indian context [41,42]. The incidence of thyroid cancer ranges from 0.06% to as high as 8.7% with females being more affected than males [43]. The commonest thyroid cancer is papillary carcinoma, followed by follicular neoplasm [41].

Imaging techniques

Radiology has become central in the diagnosis and management of patients in almost all subspecialties, but especially so in oncology [44]. Computed tomography (CT), magnetic resonance imaging (MRI), positron emission tomography (PET), and ultrasound are the various standard imaging methods for the head and neck pathologies. To find the most appropriate method, it is necessary to determine the probable structure in the affected area during examination [44].

CT scans are basically used to determine the primary location of head and neck cancer, nodal disease, and perform staging as part of the TNM (tumor [T], nodes [N], and metastasis [M]) staging. A CT scan provides a better assessment of the infrahyoid neck with less artifact activity in contrast to MRI [45]. It can also help in the evaluation of the bony cortex, cartilage involvement, and calcification and also in the evaluation of the thorax for paratracheal and upper mediastinal lymph node involvement, lung metastases, or synchronous primary lung lesions at the same time. Ultrasound offers excellent non-invasive soft tissue characterization, especially in the case of head lumps, salivary gland tumors, thyroid nodules, and geographic lymph node morphology in head and neck imaging, particularly in superficial primary sites and nodal basins. Ultrasound provides clarification for the superior image in salivary gland lesions, thyroid nodules, and lymph nodes, and it is the ideal imaging modality for guided surgical interventional procedures such as FNAC and core needle biopsy. A CT-scan biopsy is not suitable in the case of deep-seated head and neck lesions [45].

Core biopsy

A core biopsy is another way to diagnose the neck mass. A core biopsy obtains a bigger piece of tissue by using a significantly longer needle. It is well tolerated while having fewer side effects. Numerous complications including bleeding, bruising, discomfort, and infection are associated with core biopsy. In spite of this, the non-availability of a surplus amount of tissue is another problem associated with this diagnosis [46].

Open biopsy

An open biopsy is a more invasive procedure to diagnose a neck mass in which an incision is created in the skin to remove the tissue. The chances of complications with open biopsy are higher because of the invasive nature of the procedure. Several complications including anesthesia, infection, bleeding, discomfort, scarring, and nerve injury (numbness, paralysis) are associated with open biopsy [47].

FNAC

FNAC is an outpatient cytological procedure used for the diagnosis and management of suspicious nodules in the head and neck region. It is simple, comfortable, easy to administer, and a reasonably precise technique. It is safer and does not require anesthetic protocols. In addition to these, it provides quick results with no complications [48]. FNAC outcomes must be followed up to ensure effective treatment. For this purpose, a crucial distinction must be made between an insufficient specimen and one that is satisfactory but indeterminate. An incomplete specimen means that the pathologist may not have enough well-preserved lesion material to make a positive diagnosis while sufficient lesion material is available in the case of the indeterminate sample (e.g., atypical, keratin residue, or ‘neoplasm with unknown malignant potential’), but it is impossible to reach definitive conclusions regarding the diagnosis due to the intrinsic nature of the procedure [49].

Although FNAC of neck masses is very reliable due to limited false-negative diagnosis, certain patients may experience a pause in diagnosis or treatment due to a false-negative result. For this reason, while a satisfactory and negative FNAC is reassuring in certain cases, it does not rule out the requirement for more medical procedures in a patient with concerning signs and symptoms. Following an FNAC that yielded insufficient findings or diagnosis of benign pathology, a repeat FNAC could be done to ascertain malignancy. Consequently, if a patient has concerning signs and symptoms, then an open biopsy is being considered [50]. Repeat aspiration can be beneficial in certain cases of indeterminate cytology. Additionally, after an indeterminate initial outcome, a discussion with the cytopathologist can be helpful to take the decision of repeating the FNAC. Extra precautions should be taken while repeating FNAC to maximize the chances of getting a good sample for a correct diagnosis. The inclusion of ultrasound-guided FNAC has reportedly enhanced specimen adequacy; it can be useful with initial palpation-guided FNAC having limited diagnostic utility and can improve the diagnostic yield with cystic or necrotic masses by encouraging directed biopsy of the cyst's solid portion. Finally, on-site assessment by a cytopathologist may further reduce the rate of inadequacies with FNAC.

## Conclusions

Adult neck masses in the Indian population are caused by head and neck malignancy as well as infectious causes like TB. Clinical examination should be done carefully in all patients who represent a neck mass, accompanied by focused inquiries. It is important to proceed with investigations until a definite diagnosis is achieved. Suspicion of malignancy is the mainstay of investigations for all patients with a neck mass, even after CT scan and FNAC. Ancillary monitoring should be done without terminating the malignancy investigation.
